# 

**Published:** 1888-09

**Authors:** 


### THE SOUTHERN SURGICAL AND GYNECOLOGICAL ASSOCIATION.

The next meeting of this association, which takes place in Birmingham, September 11th, 12th and 13th, promises to be full of interest.

The programme, giving the subjects of papers to be read, shows several different facts. In the first place, we are astonished and pleased to see what a strong hold this young society has already taken upon the profession of the Southern States. It appears from the programme that physicians from nine different States will read papers.

In the second place, there are many good names on the programme—names of men whose scientific work is well known throughout the country.

Lastly, the subjects to be discussed are unusually varied and full of interest.

THE JOURNAL extends its heartiest wishes for the success of the meeting, and does not hesitate to predict a brilliant future for the association.

